# Response to John G. Brock-Utne. Comment on Hurley, J.C. Towards Clinical Application of Anti-endotoxin Antibodies; A Re-Appraisal of the Disconnect

**DOI:** 10.3390/toxins6041364

**Published:** 2014-04-11

**Authors:** James C. Hurley

**Affiliations:** 1 Rural Health Academic Center, Melbourne Medical School, University of Melbourne, Parkville 3010, Australia; 2 Division of Internal Medicine, Ballarat Health Services, Ballarat 3350, Australia; E-Mail: jamesh@bhs.org.au; Tel.: +61-3-5320-4322; Fax: +61-3-5320-6500; 3 Infection Control Committees, St John of God Hospital and Ballarat Health Services, Ballarat 3353, Australia

I appreciate the thoughtful comments from Dr. Brock-Utne [1] on my recent review of the disconnect between animal studies and clinical experience with anti-endotoxemia therapies [2].

Dr. Brock-Utne, the author of this commentary, has been a co-author of several important contributions in the area of human endotoxemia. Their reports that strenuous exercise causes endotoxemia in ultradistance triathlon competitors are of great interest [3,4]. They had also reported how anti-LPS antibodies change in response following the appearance of endotoxemia in these athletes. Their reports are important in that their publications are rare examples of systematic trans-species investigations of endotoxemia. Moreover, the levels of endotoxemia in human athletes they observed were not trivial with levels as high as 0.294 ng/mL. These levels were 15 times the detection limit of the assay and at levels comparable to those seen in the blood of patients in the intensive care unit with sepsis. Their reports of endotoxemia in these athletes are thought provoking and at the very least may help to explain fatalities with heat stroke [5].

However, the specific question they raise in their comment [1] as to whether anti-endotoxin therapy is feasible for Gram-negative sepsis is not simple. In my review article I point to several levels of disconnect between animal model studies and human sepsis which are problematic towards addressing the question that Dr Brock-Utne has posed. I am uncertain of the relevance of the observations of Brucellosis and its treatment published in 1948 [6] to the management of sepsis in the 21 st century [7]. Moreover, it remains unclear what relevance the infusion of *Echerichia coli* (*E. coli*) endotoxin (LPS) into laboratory animals has to the development of therapies for human sepsis. There are multiple instances of disconnect between findings in such laboratory experiments with promising anti-endotoxin agents and results of subsequent clinical studies [2]. For example, while animal model studies commonly use (*E. coli*) endotoxin, it is possible that this may have been the wrong endotoxin to study. We recently found that endotoxemia detected in association with Gram-negative sepsis with *E. coli* bacteremia does not have a significant association with mortality [8,9] although endotoxemia with non-*E. coli* bacteremia does.

To me the broader question remains enigmatic and the disconnect between animal model research and human sepsis has increased rather than decreased with time.
